# miR-122 promotes virus-induced lung disease by targeting SOCS1

**DOI:** 10.1172/jci.insight.127933

**Published:** 2021-04-08

**Authors:** Adam M. Collison, Leon A. Sokulsky, Elizabeth Kepreotes, Ana Pereira de Siqueira, Matthew Morten, Michael R. Edwards, Ross P. Walton, Nathan W. Bartlett, Ming Yang, Thi Hiep Nguyen, Sebastian L. Johnston, Paul S. Foster, Joerg Mattes

**Affiliations:** 1 Priority Research Centre GrowUpWell, Experimental and Translational Respiratory Medicine Group, School of Medicine and Public Health, University of Newcastle, Newcastle, New South Wales, Australia.; 2 Airway Disease Infection Section, National Heart and Lung Institute, Medical Research Council & Asthma UK Centre in Allergic Mechanisms of Asthma, Imperial College London, London, United Kingdom.; 3 Priority Research Centre for Healthy Lungs, University of Newcastle and Hunter Medical Research Institute, Newcastle, New South Wales, Australia.; 4 Department of Paediatric Respiratory and Sleep Medicine, John Hunter Children’s Hospital, Newcastle, New South Wales, Australia.

**Keywords:** Inflammation, Virology, Asthma, Molecular biology

## Abstract

Virus-induced respiratory tract infections are a major health burden in childhood, and available treatments are supportive rather than disease modifying. Rhinoviruses (RVs), the cause of approximately 80% of common colds, are detected in nearly half of all infants with bronchiolitis and the majority of children with an asthma exacerbation. Bronchiolitis in early life is a strong risk factor for the development of asthma. Here, we found that RV infection induced the expression of miRNA 122 (miR-122) in mouse lungs and in human airway epithelial cells. In vivo inhibition specifically in the lung reduced neutrophilic inflammation and CXCL2 expression, boosted innate IFN responses, and ameliorated airway hyperreactivity in the absence and in the presence of allergic lung inflammation. Inhibition of miR-122 in the lung increased the levels of suppressor of cytokine signaling 1 (SOCS1), which is an in vitro–validated target of miR-122. Importantly, gene silencing of SOCS1 in vivo completely reversed the protective effects of miR-122 inhibition on RV-induced lung disease. Higher miR-122 expression in nasopharyngeal aspirates was associated with a longer time on oxygen therapy and a higher rate of treatment failure in 87 infants hospitalized with moderately severe bronchiolitis. These results suggest that miR-122 promotes RV-induced lung disease via suppression of its target SOCS1 in vivo. Higher miR-122 expression was associated with worse clinical outcomes, highlighting the potential use of anti-miR-122 oligonucleotides, successfully trialed for treatment of hepatitis C, as potential therapeutics for RV-induced bronchiolitis and asthma exacerbations.

## Graphical abstract

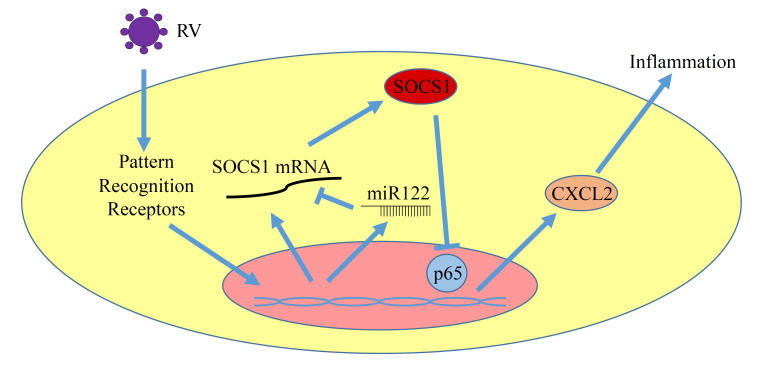

## Introduction

Virus-induced respiratory tract infections are among the most common causes for hospital admissions during childhood, manifesting as viral bronchiolitis and pneumonia in infants and asthma exacerbations and pneumonia/pneumonitis in older children. Rhinoviruses (RVs), the cause of approximately 80% of common colds, are detected in nearly half of all infants admitted to the hospital with bronchiolitis and in most children with acute asthma and viral pneumonia (1–5). While RV-induced respiratory tract infections are self-limiting in immune-competent adults, the lack of preventative and disease-modifying therapeutic strategies for bronchiolitis in infancy alone results in an increased health burden. In the US alone, costs amount to $1.73 billion (US dollars) per year.

miRNAs are small, noncoding RNA strands (approximately 22-nucleotides long) that target the 3′-untranslated region of mRNAs to inhibit posttranscriptional gene expression (6). Although miRNAs have hundreds of in silico–predicted targets, very few are validated in vitro or in vivo. Indeed, identifying mRNA targets and demonstrating the in vivo functional relevance of a miRNA-mRNA circuit is critical for understanding the role of these noncoding RNA species in health and complex disease settings. Commonly, this is inferred by showing an inverse correlation between miRNA expression and its mRNA target in vivo. However, it would be far more conclusive to suppress the expression of a miRNA of interest using oligonucleotides or gene depletion in vivo to induce a miRNA-deficient phenotype (7) and then concurrently silencing or blocking the proposed mRNA target of that miRNA to reverse the in vivo phenotype. miRNAs are well known to regulate inflammatory, antimicrobial, and antiviral immune responses in vivo (8–11). Furthermore, RV infection has been associated with differentially expressed miRNA found in extracellular vesicles of upper airway secretions from young children (12). Inflammatory signaling through NF-κB underpins the immune response to RV, and infants with bronchiolitis and RV infection have a distinct nasal airway miRNA profile associated with the upregulation of NF-κB (12). We and others have previously shown a critical role of NF-κB activation in promoting experimental RV-induced lung disease (13–15). However, the role of miRNAs in the pathogenesis of this common infection remains elusive.

## Results

**Figure 1:**
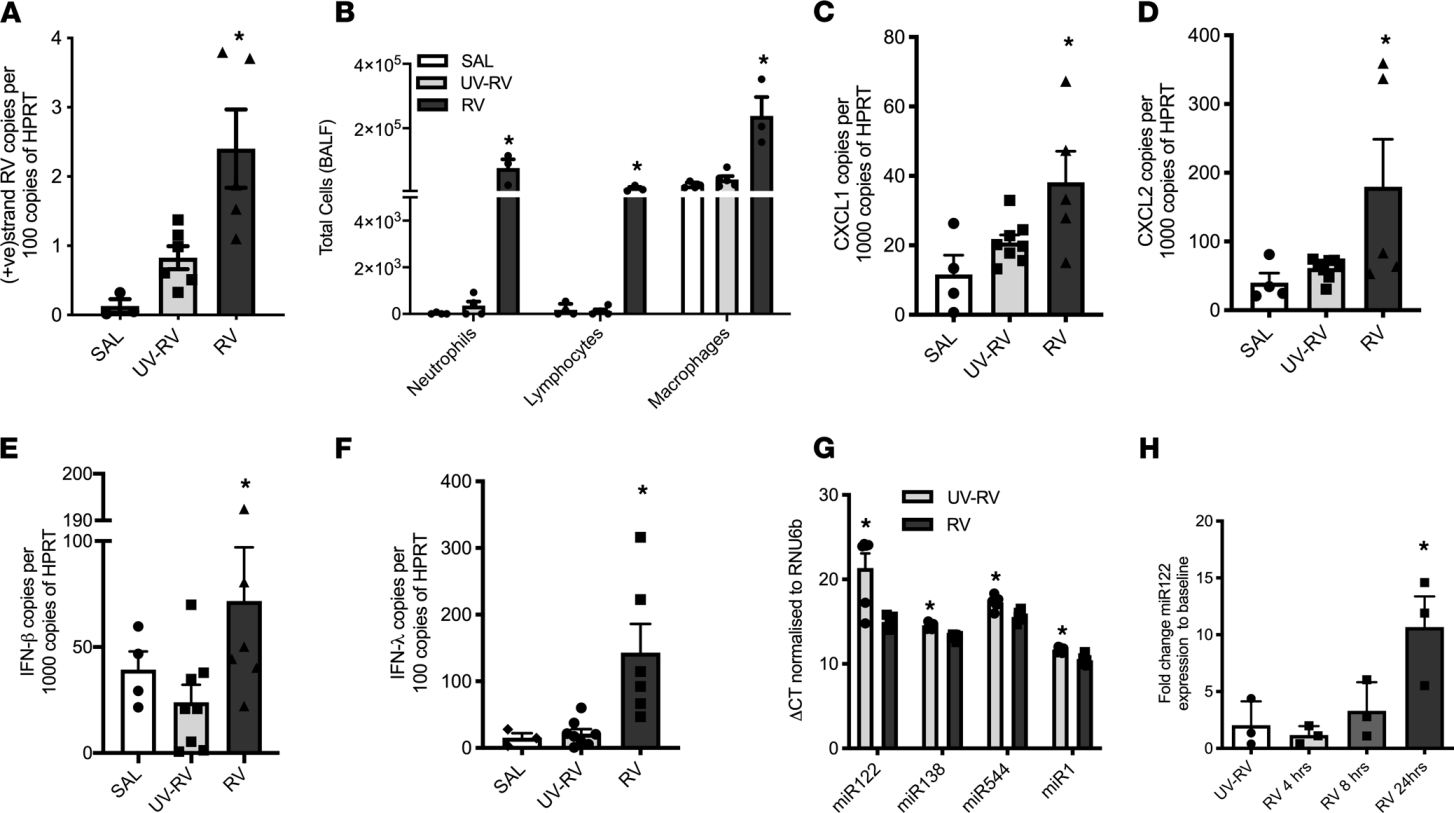
Rhinovirus-induced lung disease and miR-122 expression in infant mice. Rhinovirus (RV) replication could be detected in the lower airways at 24 hours after inoculation (**A**) (*n =* 3–6 mice per group). RV infection induced inflammation in bronchoalveolar lavage fluid (BALF) (**B**) (*n =* 3–6 mice per group) and the expression of the murine IL-8 analogs CXCL1 and CXCL2 (**C** and **D**) (*n =* 4–8 mice per group) as well as IFN-β and IFN-λ (**E** and **F**) (*n =* 4–8 mice per group). A TaqMan miRNA qPCR array, including 750 miRNAs from MIRBase v21, identified 4 miRNAs significantly upregulated in the airways of mice 24 hours after infection with RV when compared with UV controls normalized to the small nuclear–RNA (sn-RNA) control gene RNU6b (**G**) (*n =* 6 mice per group). miR-122 was induced by RV in immortalized human airways basal cells in submerged culture (**H**) (*n =* 3 independent cultures). *P < 0.05, calculated using 1-way ANOVA with multiple comparisons correcting the false discovery rate 2-stage step-up method of Benjamini, Krieger, and Yekutieli for all panels, except for **G**, where moderated 2-tailed *t* test and Westfall-Young correction for multiple testing was used. SAL, saline vehicle; UV-RV, UV-inactivated RV. Data are shown as the mean ± SEM.

**Figure 2:**
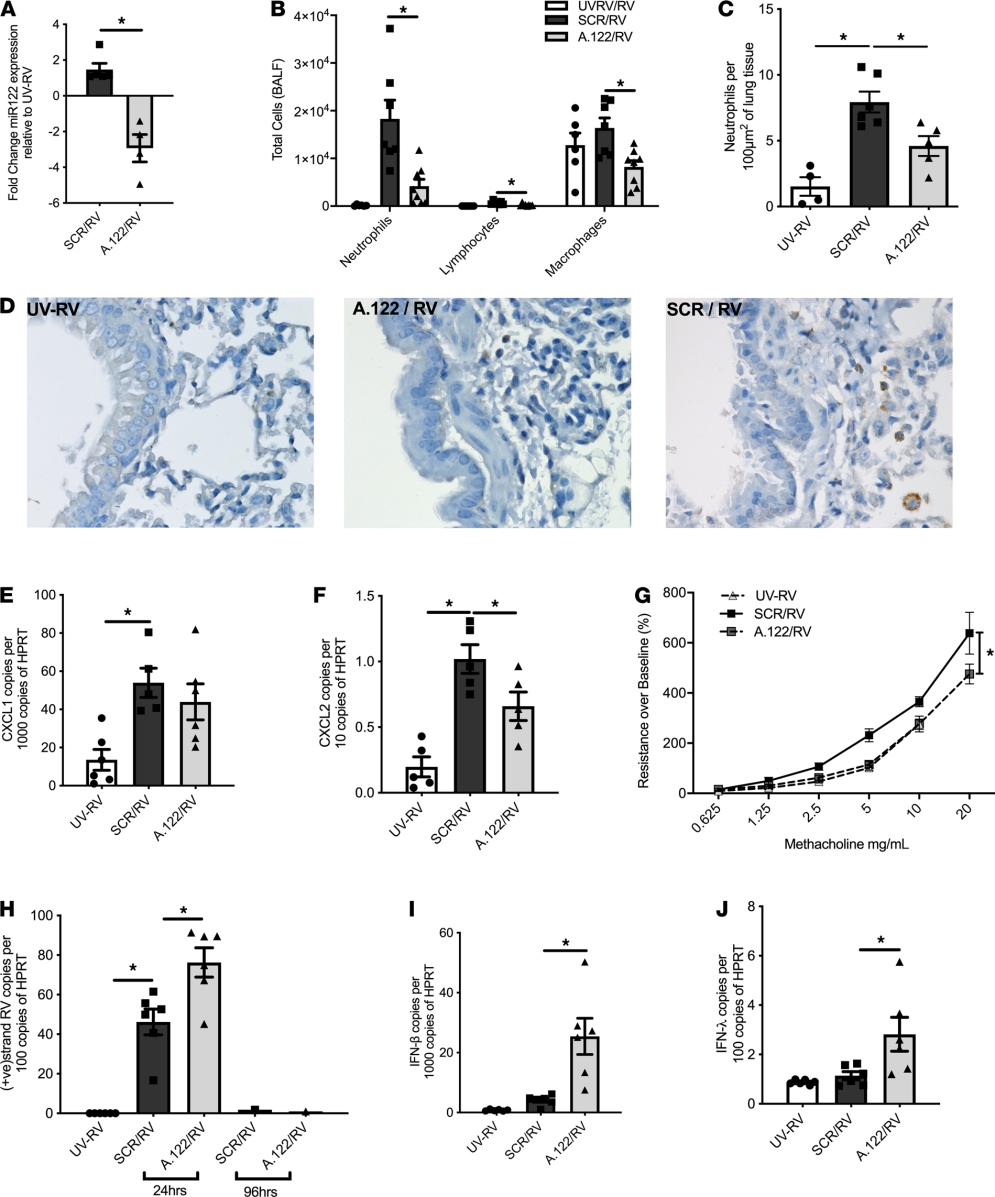
Effect of miR-122 inhibition on rhinovirus-induced lung disease in adult mice. Naive adult mice infected with RV had reduced miR-122 expression in their airways 24 hours after RV infection when treated with miR-122 antagomir (A.122/RV) as compared with mice treated with a scrambled control antagomir (SCR/RV) (**A**) (*n =* 4–5 mice per group). Mice treated with A.122/RV had reduced neutrophil influx into the BALF (**B**) (*n =* 6–8 mice per group)and fewer myeloperoxidase-positive cells (neutrophils) in the lung tissue (**C**) (*n =* 6–8 mice per group) when compared with mice treated with SCR/RV. Representative images of myeloperoxidase-positive neutrophils are shown (**D**) (*n =* 6–8 mice per group) (original magnification, ×400). Expression of mouse IL-8 analogs CXCL1 and CXCL2 was upregulated following RV infection, and CXCL2 was reduced by A.122 treatment (**E** and **F**) (*n =* 6–8 mice per group). Mice treated with miR-122 antagomir were protected from RV-induced AHR (**G**) (*n =* 6–8 mice per group). Expression of RV was increased in airways of infected mice at 24 hours and more pronounced with the inhibition of miR-122 but returned to baseline by 96 hours in all groups (**H**) (*n =* 6–8 mice per group). IFN-β and IFN-λ were increased in the airways of infected mice and more pronounced when miR-122 was inhibited (**I** and **J**). (*n =* 6–8 mice per group). *P < 0.05, calculated using 1-way ANOVA with multiple comparisons correcting the false discovery rate 2-stage step-up method of Benjamini, Krieger, and Yekutieli, except for **A** and **G**, where 2-tailed *t* test and 2-way ANOVA were used, respectively. UVRV, UV-inactivated RV. Data are shown as the mean ± SEM.

To gain insights into the role of miRNA in the pathogenesis of RV-induced lung disease such as bronchiolitis, we inoculated 7-day-old infant mice with RV or UV-inactivated RV as a control via the nasal route. This resulted in detectable RV replication in the lower airways, influx of inflammatory cells into the bronchoalveolar space, upregulated expression of the neutrophil chemoattractants CXCL1 and CXCL2 in the lung, and the production of innate antiviral IFN production (Figure 1, A–F). Next, we determined miRNA expression in lung tissues using a PCR-based miRNA array and identified miRNA 122 (miR-122) as the most significantly upregulated miRNA in the lungs of infant mice 24 hours after RV inoculation (Figure 1G). miR-122 was also overexpressed in human airway epithelial cells 24 hours after RV exposure in vitro (Figure 1H). To therapeutically modulate miR-122 expression in the lungs via the airway route, we infected mice with RV and suppressed miR-122 expression levels (Figure 2A) by employing miR-122 antagomirs, which are chemically modified oligonucleotides exactly complementary to the single-stranded miR-122 that has been associated with an RNA-silencing complex. Targeting miR-122 with antagomirs in vivo resulted in reduced influx of inflammatory cells into the bronchoalveolar space (Figure 2B) and less neutrophilic inflammation in lung tissue as compared with treatment with scrambled nonsense oligonucleotides (Figure 2, C and D). Inhibition of miR-122 was associated with reduced CXCL2 but not CXCL1 expression (Figure 2, E and F) and the abolishment of RV-induced airway hyperreactivity (AHR) (Figure 2G). Interestingly, a reduced inflammatory response in the lung was associated with increased RV replication (Figure 2H) and innate IFN production in the lower airways (Figure 2, I and J).

**Figure 3:**
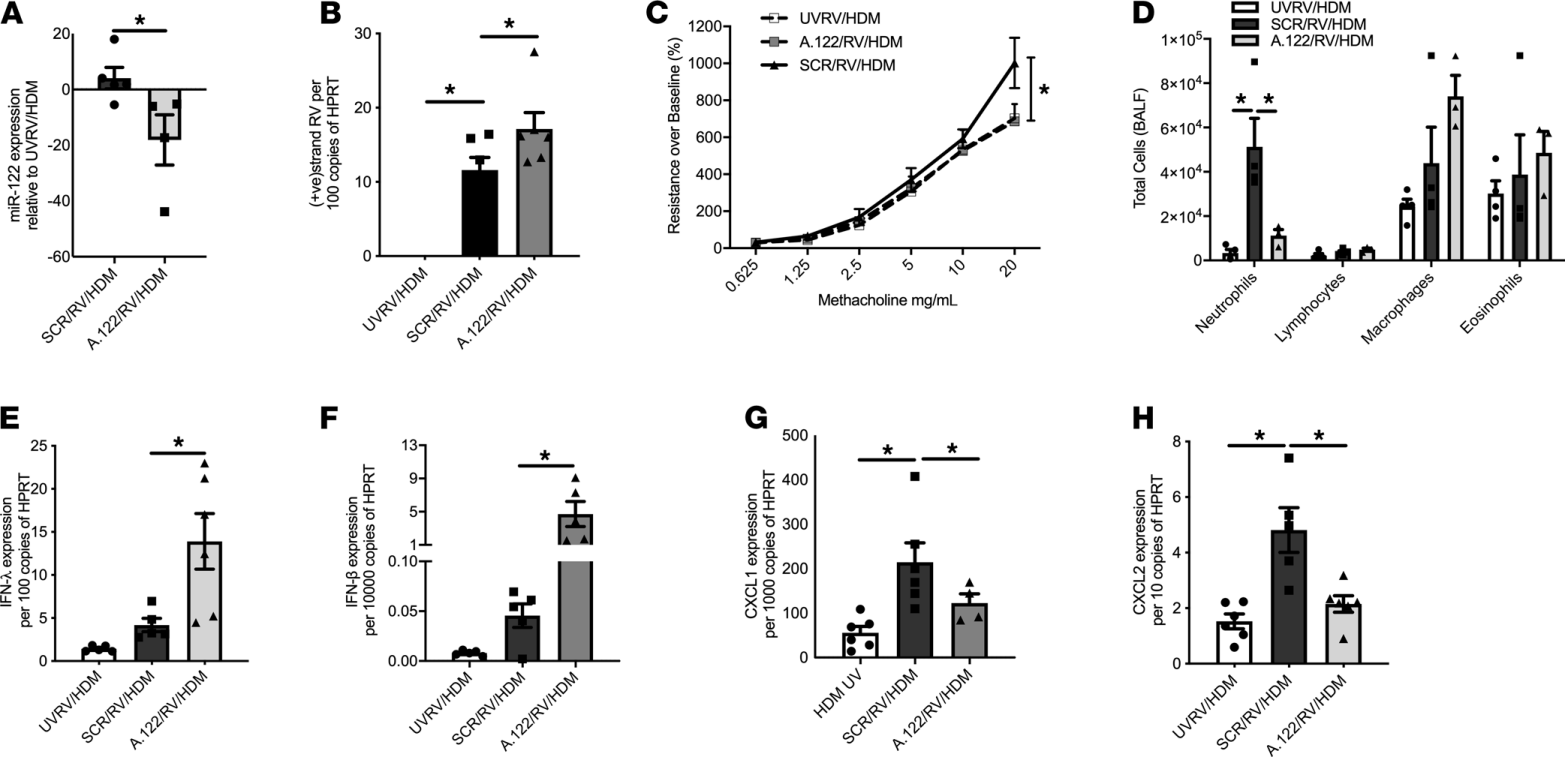
Effect of miR-122 inhibition on rhinovirus-induced exacerbation of allergic lung disease in adult mice. House dust mite (HDM) sensitized and challenged mice infected with RV had increased expression of miR-122 in the airways (SCR/RV/HDM), which was ameliorated by antagomir treatment targeting miR-122 (A122/RV/HDM) (**A**) (*n =* 4–6 mice per group). Increased RV replication was observed in the airways in mice where miR-122 was inhibited (**B**) (*n =* 4–6 mice per group). Mice treated with miR-122 antagomir were protected from RV-induced AHR (**C**) (*n =* 4–6 mice per group) and had reduced neutrophil influx (**D**) into the BALF when compared with mice treated with a scrambled control antagomir (SCR/RV/HDM) (*n =* 3–4 mice per group). Expression of IFN-β and IFN-λ in the airways was further increased by inhibition of miR-122 (A122/RV/HDM) (**E** and **F**) (*n =* 4–6 mice per group). CXCL1 and CXCL2 were also increased by RV and reduced by inhibition of miR-122 (**G** and **H**) (*n =* 4–6 mice per group). *P < 0.05, calculated using 1-way ANOVA with multiple comparisons correcting the false discovery rate 2-stage step-up method of Benjamini, Krieger, and Yekutieli, except for **A** and **C**, where 2-tailed *t* test and 2-way ANOVA were used, respectively. UVRV, UV-inactivated RV. Data are shown as the mean ± SEM.

RV infection is the most common cause for asthma attacks in childhood (3). In order to model a RV-induced asthma exacerbation, we challenged adult mice repeatedly with house dust mite allergen to induce allergic airway disease and then superimposed a RV infection (13). Similar to results found in nonallergic mice, miR-122 inhibition limited cell influx of neutrophils into the bronchoalveolar space, which was associated with reduced CXCL2 expression, abolishment of AHR, and increased RV replication and innate IFN production (Figure 3). These results demonstrate that RV-induced miR-122 promotes an influx of neutrophils into the lung along with AHR; together, these are associated with reduced RV replication and innate IFN expression in the presence and in the absence of allergic airway disease.

**Figure 4:**
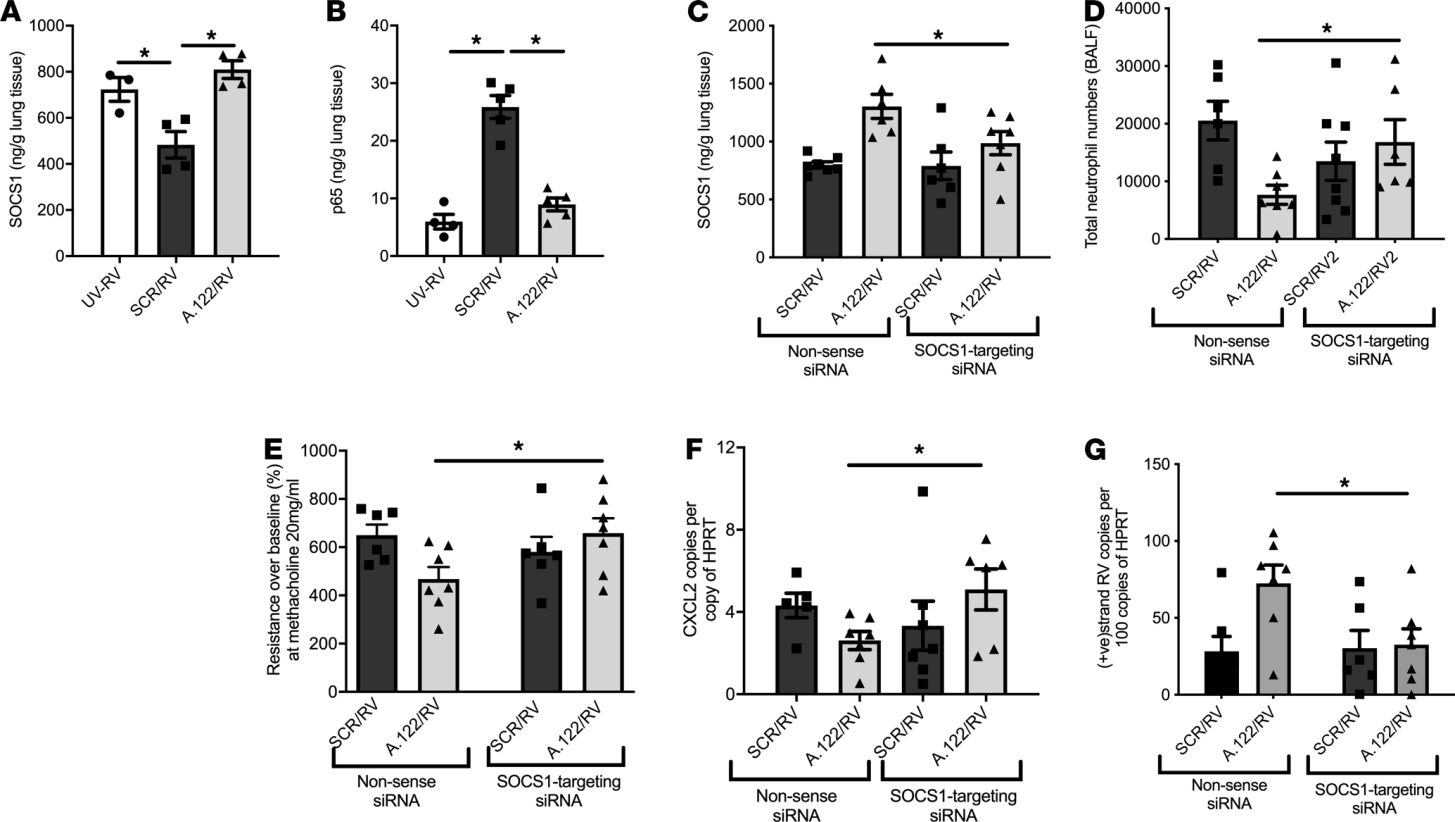
Modulation of rhinovirus-induced lung disease by SOCS1 and miR-122 inhibition in adult mice. Naive BALB/c mice were treated with antagomirs targeting miR-122 (A.122) or scrambled control (SCR). RV infection reduced levels of SOCS1 in the lungs, which was reversed by miR-122 inhibition (**A**) (*n =* 3–5 mice per group). The NF-κB subunit p65 was upregulated by RV, and this upregulation was reversed by miR-122 inhibition (**B**) (*n =* 3–5 mice per group). In separate experiments, mice also received siRNA-targeting SOCS1 or a nonsense sequence siRNA as control (NON) along with A.122 or SCR before being infected with RV (**C**). The effects of miR-122 inhibition on neutrophil influx into the bronchoalveolar space (**D**), AHR (**E**), CXCL2 (**F**), and RV replication (**G**) were all neutralized by inhibition of SOCS1 (*n =* 6–8 mice per group). *P ≤ 0.05, calculated using 1-way ANOVA with multiple comparisons correcting the false discovery rate 2-stage step-up method of Benjamini, Krieger, and Yekutieli, except for **C**–**G**, where 2-tailed *t* test was used to determine the effects of SOCS1 inhibition in A.122-treated mice. UV-RV, UV-inactivated RV. Data are shown as the mean ± SEM.

Suppressor of cytokine signaling 1 (SOCS1) is an in silico– and in vitro–validated target of miR-122 (16); SOCS1 operates as a ubiquitin ligase that causes the polyubiquitination and proteasomal degradation of nuclear p65,which terminates the expression of NF-κB–inducible genes (17) and the suppression of RV-induced innate IFN production (18). The role of SOCS1 in RV infection renders it the most plausible in vivo target of miR-122 to mediate its actions on a posttranslational level. This hypothesis was supported by increased SOCS1 and reduced p65 protein levels in lungs of RV-infected mice treated with miR-122 antagomirs as compared with those treated with a scrambled oligonucleotide (Figure 4, A and B). In order to demonstrate unequivocally the mechanistic relevance of miR-122–SOCS1 target interaction in vivo for the expression of RV-induced lung disease, we treated mice with miR-122 antagomirs (or scrambled oligonucleotides) to increase the transcript levels of the miR-122 target SOCS1. Concurrent with this approach, we also silenced SOCS1 expression with inhibitory siRNA and compared the effects to treatment with a scrambled nonsense siRNA (Figure 4C). Importantly, the effects of miR-122 inhibition on neutrophil influx into the bronchoalveolar space (Figure 4D), AHR (Figure 4E), CXCL2 expression (Figure 4F) and RV replication (Figure 4G) were all reversed by inhibition of SOCS1. Thus, miR-122 mediates its proinflammatory and antiviral effects in RV-induced lung disease at a posttranslational level via reduced SOCS1 in vivo.

**Figure 5:**
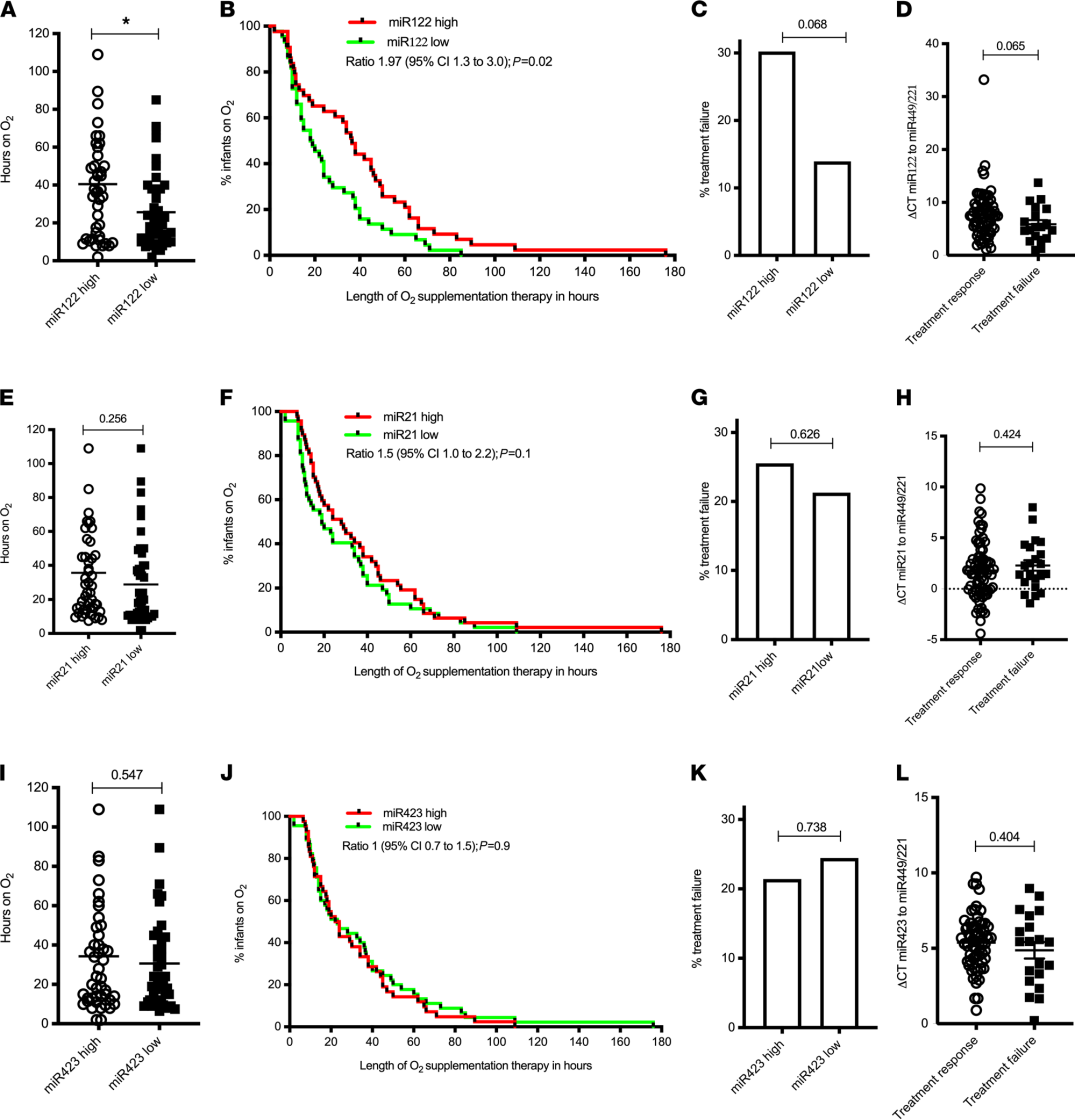
miRNA 122 in nasopharyngeal aspirates from infants admitted to the hospital with moderately severe bronchiolitis. Infants with high miR-122 expression spent more hours on oxygen during their admission (**A** and **B**) and failed treatment more often (**C**). Infants who failed treatment trended toward increased detectable levels of miR-122 expression (**D**). Infants with higher levels of control miRNAs miR-21 and miR-423 had no difference in time on oxygen (**E**, **F**, **I**, and **J**, respectively) and no differences in the rate of treatment failure (**G** and **K**). Those that failed treatment had no difference in the expression of either miR-21 (**H**) or miR-423 (**L**) (*n =* 87). *P ≤ 0.05, calculated using Mann-Whitney test, except for **B**, **F**, and **J**, where Gehan-Breslow-Wilcoxon test was used to compare survival curves.

Lower airway specimens in acute RV infection are difficult to obtain in nonventilated patients. We therefore quantified miR-122 expression in nasopharyngeal aspirates (NPAs) in a tertiary outcome analysis of NPA samples collected from 87 hospitalized infants with moderately severe bronchiolitis from a cohort of 202 infants who participated in a controlled trial in which they were randomized to either low- or high-flow oxygen supplementation therapy (1). There was no difference in baseline characteristics between the original cohort and the 2 subgroups of infants from whom NPA supernatant or cell pellets were available for miR-122 expression profiling, with 52 of 87 individuals being conserved across both groups (Supplemental Table 1; supplemental material available online with this article; https://doi.org/10.1172/jci.insight.127933DS1). In the trial, the primary outcome was time on oxygen supplementation therapy, and secondary outcomes included treatment failure requiring escalation of care, such as transfer to an intensive care unit. Notably, infants with high miR-122 expression in NPA supernatants, defined as miR-122 expression above the median of the cohort, were on oxygen therapy for a significantly longer time, which was the primary outcome of the trial in which this study was nested (Figure 5, A and B). Furthermore, a statistical trend toward more infants with high miR-122 expression failing their allocated treatment compared with infants with low miR-122 expression (Figure 5C) was observed. Conversely, those children who failed their treatment also showed a trend toward higher miR-122 expression in their NPAs (Figure 5D). Importantly, miR-21 and miR-423, used as controls, were not associated with any of these outcomes (Figure 5, E–L). In addition, statistically significant associations were observed between miR-122 expression and clinical outcomes in the subcohort with NPA cell pellets available (Supplemental Figure 1). These associations were seen across the cohort as a whole, with approximately one-half of the infants positive for RV and the other half positive for respiratory syncytial virus. Thus, miR-122 expression is associated with clinically important outcomes in infants admitted to the hospital with moderately severe bronchiolitis due to RV or respiratory syncytial virus infection. Future studies are required to elucidate the potential of monitoring miR-122 expression as a predictive biomarker for disease severity.

## Discussion

miR-122 was initially thought to be a tissue-specific miRNA, representing 70% of the total miRNA population in the liver (19). In the liver, hepatitis C virus (HCV) may exploit miR-122 for viral replication, whereby miR-122 binds to the 5′-noncoding region of viral genomic RNA to promote viral stability and translation of viral proteins (20, 21). Our studies show an unexpected role of miR-122 in the lung and indicate its interaction with the host-derived antiviral response to promote neutrophilic inflammation in a SOCS1-dependent manner. Higher numbers of neutrophils in the airways are related to virus load in the lung, clinical severity of infection, and AHR in acute experimental RV infection of healthy individuals and subjects with asthma (22). Paradoxically, neutrophils may also play an important role in innate immune responses to clear virus (23). This may explain higher RV replication upon miR-122 inhibition at 24 hours of infection, which was not evident at later time points (Figure 2H and Figure 3B and data not shown). While inhibition of miR-122 reduced neutrophil influx, the expression of the chemoattractant CXCL2 but not CXCL1 was reduced. This complex regulatory interplay between CXCL1 and CXCL2 in neutrophilic chemotaxis, and the apparent paradox between protective and pathologic effects of neutrophils, requires further studies. Those may include investigations of the therapeutic potential of targeting neutrophil extracellular traps, which contribute to the severity of RV-induced asthma exacerbations (24, 25). The clinical relevance of miR-122 in the modulation of virus-induced respiratory tract infections was supported by an association between higher miR-122 expression in NPA and longer time on oxygen treatment and trends with higher rates of treatment failure in infants with bronchiolitis. We, therefore, propose that targeting of miR-122 may be of therapeutic benefit in RV-induced lung disease. As bronchiolitis in early life is a strong risk factor for the development of asthma (26), disease-modifying treatments may have the potential to ameliorate asthma in susceptible children. These hypotheses are testable, as anti-miR-122 oligonucleotides have been trialed in phase I and II clinical studies involving subjects with HCV infection. One administration of a hepatocyte-targeted N-acetylgalactosamine–conjugated anti-miR-122 oligonucleotide was well tolerated and resulted in substantial HCV viral load reduction (27). The use of Miravirsen, a locked nucleic acid–modified DNA phosphorothioate antisense oligonucleotide for miR-122, showed prolonged dose-dependent reductions in HCV RNA levels (28). All together, we have shown a role of miR-122 in promoting RV-induced lung disease that has the potential to be therapeutically and diagnostically exploitable in the future.

## Methods

Additional information can be found in Supplemental Methods.

### Study approval.

All mouse experiments were approved by the Animal Care and Ethics Committee of the University of Newcastle. All patient samples were collected after written informed consent from the parent. The study was approved by the Human Research Ethics Committees of the Hunter New England Local Health District, Newcastle, New South Wales, Australia, and the University of Newcastle.

## Author contributions

AMC and LAS designed and performed the experiments, analyzed data, and drafted the manuscript. EK coordinated the clinical trial and provided samples. APDS, MM, MY, and THN assisted with assays and technical expertise. MRE, RPW, NWB, and SLJ provided the RV for the animal models and expertise. PSF supervised experiments and interpreted data. JM coordinated, designed and supervised experiments and the clinical trial; interpreted clinical and experimental data; and drafted manuscript versions. All authors reviewed and edited the manuscript.

## Supplementary Material

Supplemental data

## Version 1 04/08/2021

Electronic publication
