# Supplementary material for: miR-122 promotes virus-induced lung disease by targeting SOCS1
Source: JCI Insight. 2021 Apr 8;6(7):e127933. doi: 10.1172/jci.insight.127933 (PMC8119205; doi:10.1172/jci.insight.127933)
Supplement: Supplemental data [file jciinsight-6-127933-s116.pdf]

Online Supplement

Methods

Cell culture

Immortalized human airway basal cells(1) were grown in bronchial epithelial cell growth medium (BEGM, Clonetics). After one passage, cells were seeded 2 x 10^5 per ml onto 12-well plate, cultured until 80% confluence, serum-starved for 24 hours, inoculated with RVA1 (multiplicity of infection of 2) and re-cultured for up to 24 hours in serum-free BEGM media as previously described(2).

Mice

Wild type BALB/c mice were obtained from Australian Bioresources (Moss Vale, Australia). Mice were housed in the Bioresources facility located within the HMRI building and had ad libitum access to food and water.

RV infection and induction of allergic airways disease

HDM was used to induce allergic airways disease in mice as previously described(3). Briefly, mice were sensitized with 50µg of HDM intranasally under isoflurane anesthesia three times over three days. Mice were left to rest for 12 days before being challenged with 5µg of HDM intranasally for four days. 24hrs post final HDM challenge, HDM allergic and naïve non-allergic mice were administered with infective or UV-inactivated RVA1 (2.5x10^6 median tissue culture infective dose) intranasally under isoflurane anesthesia in 50ul of virus inoculum in adult mice and 10ul in 7-day old mice (4). Mice were then assessed for airways inflammation and hyperactivity 24 hours post infection.

MiR122 and SOCS1 silencing

24 or 48 hours prior to infection, mice were administered antagomirs targeting miR122 (A.122) (CAAACACCAUUGUCACACUCCA) or scramble control (SCR) (UUGUACUACACAAAAGUACUG) (50µg/50µl) intranasally under isoflurane anesthesia. For the silencing of SOCS1, siRNAs specific for SOCS1 or nonsense control (NON) (Ambion, Life Science, Australia) were administered to mice 24 hours after antagomir administration and 24 hours prior to infection.

AHR measurements

Mice were anesthetized using ketamine and xylazine (Illum) prior to being mechanically ventilated and assessed for lung resistance and dynamic compliance (Buxco). AHR was induced in mice through increased doses of nebulized methacholine (Sigma) and normalized as percentage over baseline as previously described(2).

Bronchoalveolar lavage fluid (BALF)

Euthanized mice had their tracheas cannulated and flushed with 1 ml of HBSS (via syringe) for bronchoalveolar cell collection. For cell enumeration and differentiation, total number of cells per lavage was determined prior to being cytospun onto a microscope slide. Cells from BALF were stained using May-Grunwald and Giemsa solution and differential counts were performed blinded using light microscopy.

Isolation of RNA and gene expression

Whole lung and airway wall tissue (isolated by blunt dissection as previously described (2)) were immersed in TRIzol® (Ambion, Life Technologies, Mulgrave, Australia) and homogenized using a stick blender (TissueTearor). Cells were immersed in TRIzol® at collection. RNA was then extracted in accordance to the manufacturer's instructions and resuspended in nuclease free water.

mRNA was then reverse transcribed using Bioscript (Bioline, Alexandria, Australia) into cDNA and gene expression was assessed by quantitative PCR (Eppendorf, Hamburg, Germany) using SYBR green (Life Technologies, Mulgrave, Australia) and primers targeting specific genes (Table 2).

Taqman® microRNA array

RNA samples were reverse transcribed using the TaqMan array kit according to the manufacturer's instructions using Megaplex™ RT primers and Multiscribe™ Reverse Transcriptase (Applied Bioscience). RT product was diluted with TaqMan® Universal PCR Master Mix (No AmpErase® UNG, 2x), and dispensed into each port of the TagMan miRNA MicroArray card v3 (Applied Biosystems). TaqMan Low Density Array thermo-cycling conditions were used on a Viia7 qPCR machine (Applied Biosystems). Gene array analysis was determined using Genespring GX (Agilent) software with the relative expression of each miRNA normalized to the RNU6b small nuclear (sn)-RNA control gene.

Immunohistochemistry myeloperoxidase stain for neutrophils

Formalin-fixed lung sections were blocked with 25% (vol/vol) sheep serum (SAFC Biosciences) for 2 h before being incubated with Myeloperoxidase (MPO) antibody (R&D Systems, Minneapolis, MN, USA, 1:200) followed by a secondary Biotin conjugated antibody (Santa Cruz Biotechnology, cat no. sc-2042, 1:2,000). Pierce High Sensitivity Streptavidin-HRP (Thermo Fisher Scientific) was used for development, prior to counterstaining with hematoxylin (Sigma).

SOCS1 ELISA and NFκB activity analysis

Protein was extracted from snap-frozen murine lung samples by immersing lungs in IC#10 diluent (EGTA, EDTA, NP-40 alternative, HEPES and water) supplemented with Leupeptin

(2.5mg/mL), Pepstatin (1.25mg/mL), Aprotinin (1.1mg/mL) and PMSF) prior to homogenization. Homogenized protein samples were then assessed for SOCS1 protein by ELISA (MyBioSource, Preston, Victoria) and normalized to total lung weight (mg). Alternatively, total protein was determined for each sample using Bicinchoninic Acid Assay (Thermo Fisher Scientific, Australia) and 40ug of total protein per sample was allocated for TransAM Transcription Factor p65 assay kit (Active Motif, Karrinyup, Western Australia).

RNA isolation from NPA

Nasopharyngeal Aspirates (NPA) were collected from children presenting at John Hunter Children's Hospital and enrolled in the High-Flow Warm Humidified Oxygen Randomized controlled trial as previously described[1]. Samples were stored at 4°C prior to being spun at 4000 rpm for 10 mins. The supernatant was then decanted into a separate tube and the cell pellet immersed in TRIzol® (Ambion, Life Technologies, Mulgrave, Australia). Both the Pellet and supernatant we stored at -80°C until RNA extraction. Pellet RNA was then extracted in accordance to the manufacturer's instructions and resuspended in nuclease free water. Supernantant RNA was extracted using NucleoSpin miRNA Plasma, Mini kit for circulating miRNA (Machery-Nagel, Dueren, Germany). RNA was resuspended in nuclease free water. Predesigned TaqMan primers and probes (Applied Biosystems, Australia) were employed to quantify miR122 or the endogenous controls Sno202, miR-221 or miR-449 according to the manufacturers protocol.

Statistical analysis

The statistical differences between groups were determined by employing one-way ANOVA with multiple comparisons correcting the false discovery rate two-stage step-up method of Benjamini, Krieger and Yekutieli, two-tailed *t-tests,* Mann-Whitney tests or two-way analysis of variance (for AHR data) using Graphpad Prism 7 where appropriate. Analysis of the TaqMan

qPCR miRNA microarray card was conducted using Genespring GX with a moderated T-test and Westfall-Young correction for multiple testing. Gehan-Breslow-Wilcoxon test was used to compare survival curves.

Study approval

All mouse experiments were approved by the Animal Care and Ethics Committee of the University of Newcastle. All patient samples were collected with the study was approved by the Human Research Ethics Committees of the Hunter New England Local Health District and the University of Newcastle, NSW[1].

Supp Figure 1: miRNA 122 was quantified using qPCR in nasopharyngeal aspirate pellets from infants admitted to hospital with moderately severe bronchiolitis. Infants with high miR122 expression spent more hours on oxygen during their admission **(A, B)** and more often failed treatment **(C).** Infants who failed treatment had higher detectable levels of miR122 expression **(D)**(n=87). * $p \leq 0.05$ calculated using Mann-Whitney test except for (B) where Gehan-Breslow-Wilcoxon test was used to compare survival curves.

Supp Figure 1

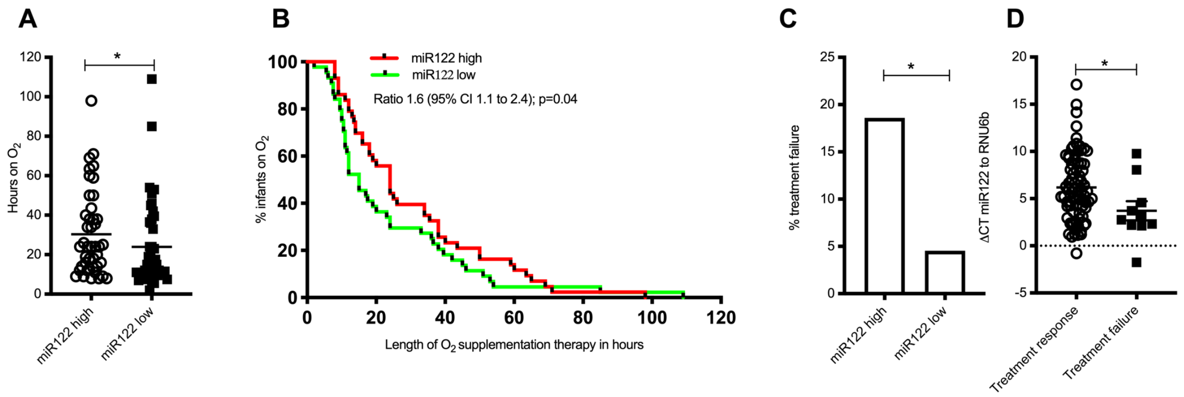

Supplement Table 1: Participant Characteristics

	Sub cohort with NPA pellet miRNA data	Sub cohort with NPA supernatant miRNA data	Original RCT cohort *(5)*
Number	87	87	202
Male	58 (67%)	59 (68%)	138 (68%)
Female	29 (33%)	28 (32%)	64 (32%)
Median age (months)	6 (3-10)	6 (3-10)	6 (3-10)
Ever breastfed	61 (70%)	57 (66%)	139 (69%)
Day of illness	4 (3-5)	4 (3-5)	4 (3-5)
Weight (kg)	7.85 (5.9-10)	7.6 (5.7-10.1)	7.96 (6-9.7)
Baseline M-WCAS	2.46 (1.75-3)	2.50 (2-3)	2.56 (2-3)
Age (stratified)			
≤1 month	14 (16%)	17 (20%)	24 (12%)
1·1–12 months	58 (67%)	54 (62%)	145 (72%)
12·1–24 months	15 (17%)	16 (18%)	33 (16%)
Gestational age at birth			
Extremely premature (≤28 weeks)	5 (6%)	3 (3%)	7 (3%)
Premature (28 weeks and 1 day to 36 weeks and 6 days)	20 (23%)	19 (22%)	30 (15%)
Term (≥37 weeks)	62 (72%)	64 (74%)	165 (82%)
Treatment Failure	10 (12%)	19 (22%)	47 (23%)
Time on Oxygen	19 (11-38)	24 (11.5-45.5)	23.5 (11.5-45.7)
Viral infections			
Concurrent infections			
0	3 (3%)	5 (6%)	6 (3%)
1	58 (67%)	56 (64%)	142 (70%)
2	22 (25%)	20 (23%)	46 (23%)
3	3 (2%)	4 (5%)	7 (3%)
4	0 (0%)	0 (0%)	1 (0%)
RSV	52 (60%)	46 (53%)	117 (58%)
Rhinovirus	38 (44%)	42 (48%)	93 (46%)
Adenovirus	13 (15%)	9 (10%)	19 (9%)
Human metapneumovirus	6 (7%)	6 (7%)	16 (8%)
Parainfluenza 2	1 (1%)	1 (1%)	1 (<1%)
Parainfluenza 3	5 (6%)	4 (5%)	8 (4%)
Influenza A	0 (<1%)	2 (2%)	2 (1%)
Influenza B	0 (<1%)	0 (0%)	1 (<1%)

Supplement Table 2: Primer list and Sequence

Gene	Sequence (Forward & Reverse)
HPRT	F: 5'-AGGCCAGACTTTGTTGGATTTGAA-3',<br>R: 5'-CAACTTGCGCTCATCTTAGGCTTT-3'
RVA1	F: 5'-AGTCCTCCGGCCCCTGAATG-3',<br>R: 5'-AAAGTAGTYGGTCCCATCCGC-3'
CXCL1	F: 5'-TGCACCCAAACCGAAGTCAT-3',<br>R: 5'-TTGTCAGAAGCCAGCGTTCAC-3'
CXCL2	F: 5'-AGTGAACTGCGCTGTCAATGC-3',<br>R: 5'-AGGCAAACTTTTTGACCGCC-3'
IFN-λ	F: 5'-GAGAAGGACCTGAGGTGCAG-3',<br>R: 5'-CTGTGGCCTGAAGCTGTGTA-3'
IFN-β	F: 5'-AAGAGTTACACTGCCTTTGCCATC -3',<br>R: 5'-CACTGTCTGCTGGTGGAGTTCATC-3'
SOCS-1	F: 5'-AGTTCCTTCCCCTTCCAGAT-3',<br>R: 5'-ATGAGGTCTCCAGCCAGAAG-3'

1. Walters M, S. Gomi, K. Ashbridge, B. Moore, M, A, S. Arbelaez, V. Heldrich, J. Ding, BS. Rafi, S. Staudt, M, R. Crystal, R, G. . Generation of a human airway epithelium derived basal cell line with multipotent differentiation capacity. *Respiratory Research.* 2013;14(135).
2. Collison A, Hatchwell L, Verrills N, Wark PA, de Siqueira AP, Tooze M, et al. The E3 ubiquitin ligase midline 1 promotes allergen and rhinovirus-induced asthma by inhibiting protein phosphatase 2A activity. *Nat Med.* 2013;19(2):232-7.
3. Girkin JL, Hatchwell LM, Collison AM, Starkey MR, Hansbro PM, Yagita H, et al. TRAIL signaling is proinflammatory and proviral in a murine model of rhinovirus 1B infection. *Am J Physiol Lung Cell Mol Physiol.* 2017;312(1):L89-L99.
4. Bartlett NW, Walton RP, Edwards MR, Aniscenko J, Caramori G, Zhu J, et al. Mouse models of rhinovirus-induced disease and exacerbation of allergic airway inflammation. *Nat Med.* 2008;14(2):199-204.
5. Kepreotes E, Whitehead B, Attia J, Oldmeadow C, Collison A, Searles A, et al. High-flow warm humidified oxygen versus standard low-flow nasal cannula oxygen for moderate bronchiolitis (HFWHO RCT): an open, phase 4, randomised controlled trial. *Lancet.* 2017;389(10072):930-9.
